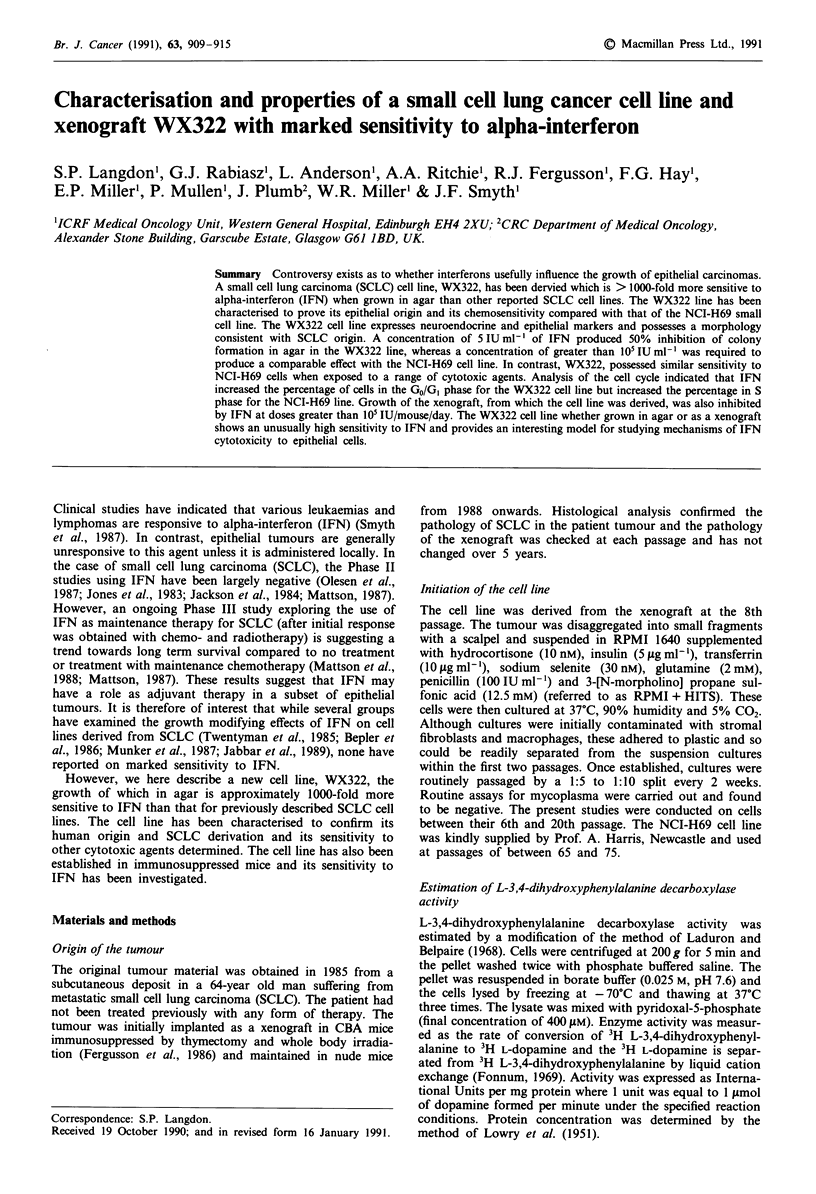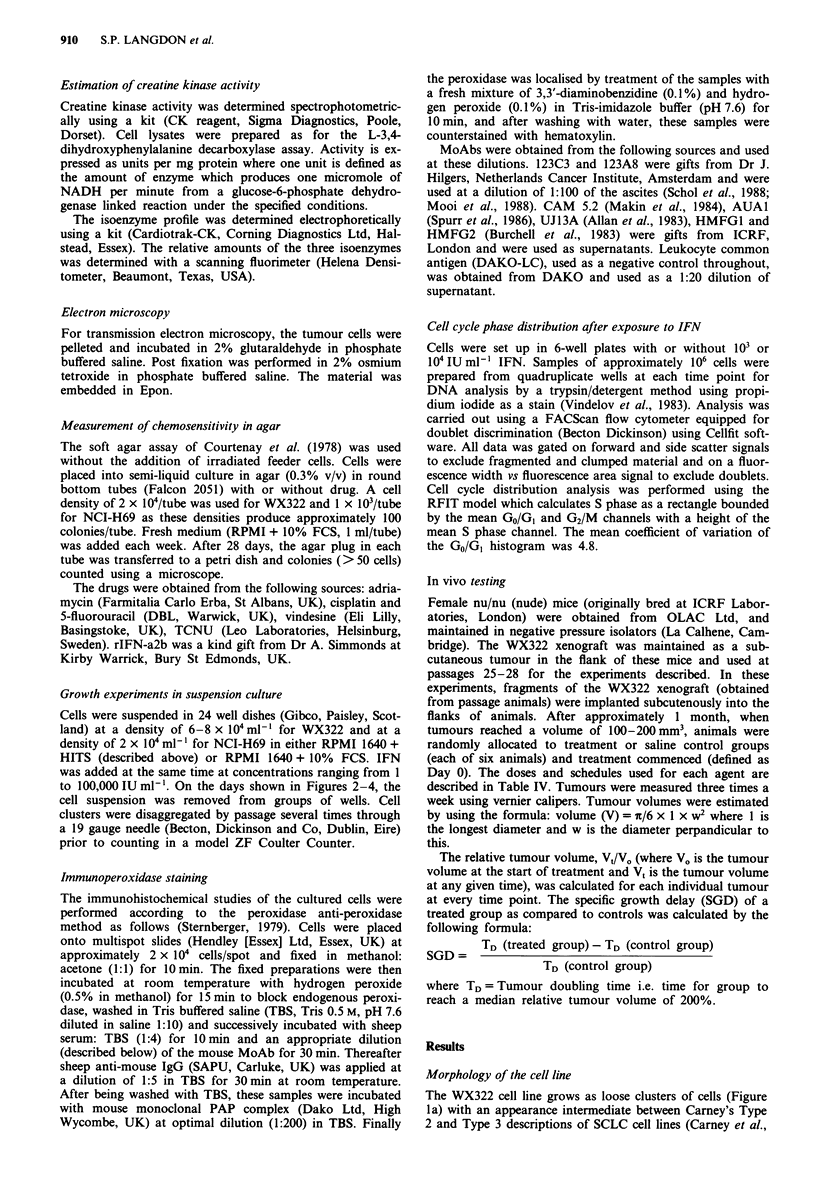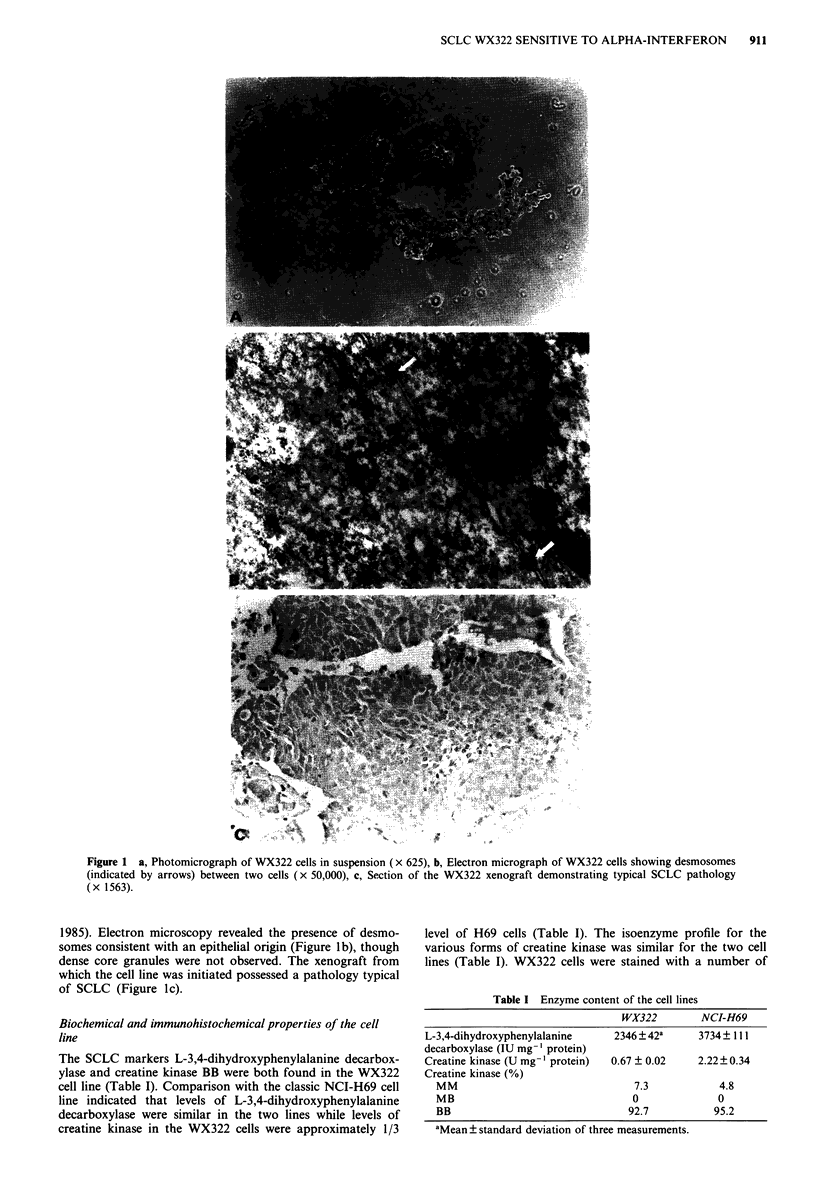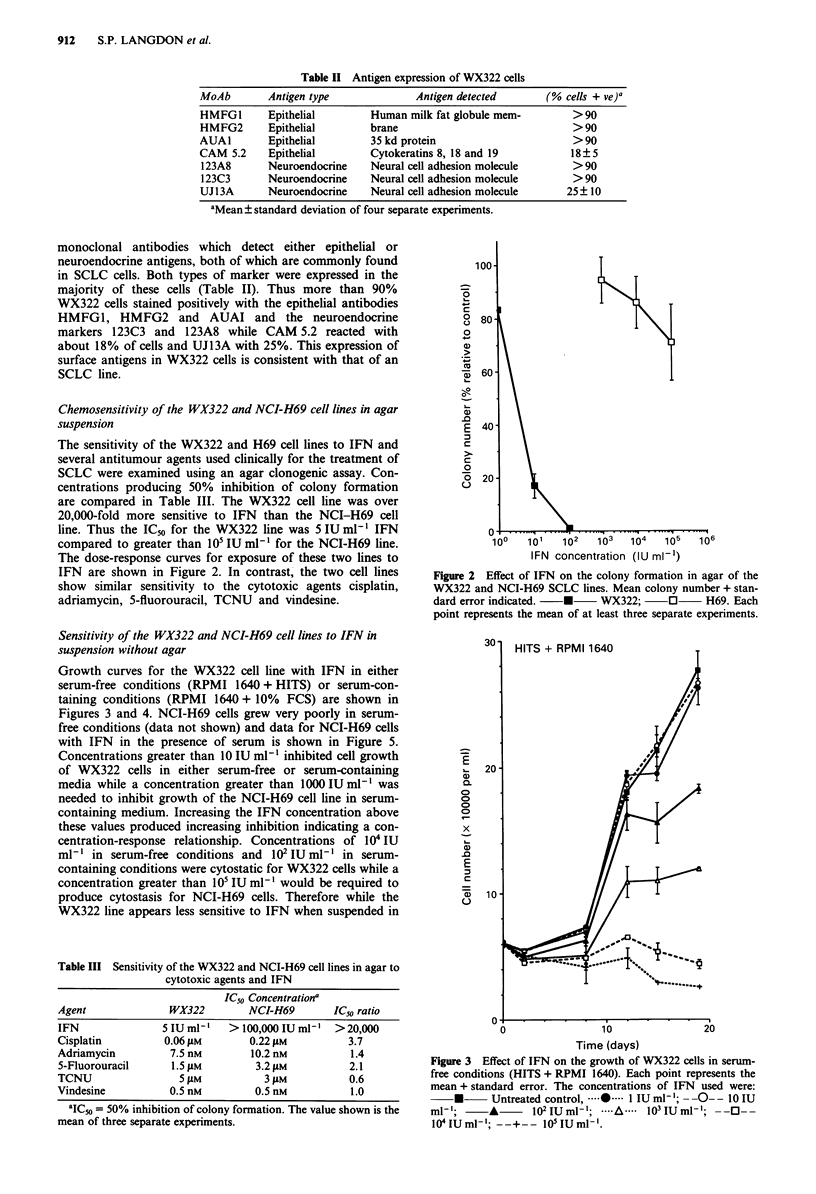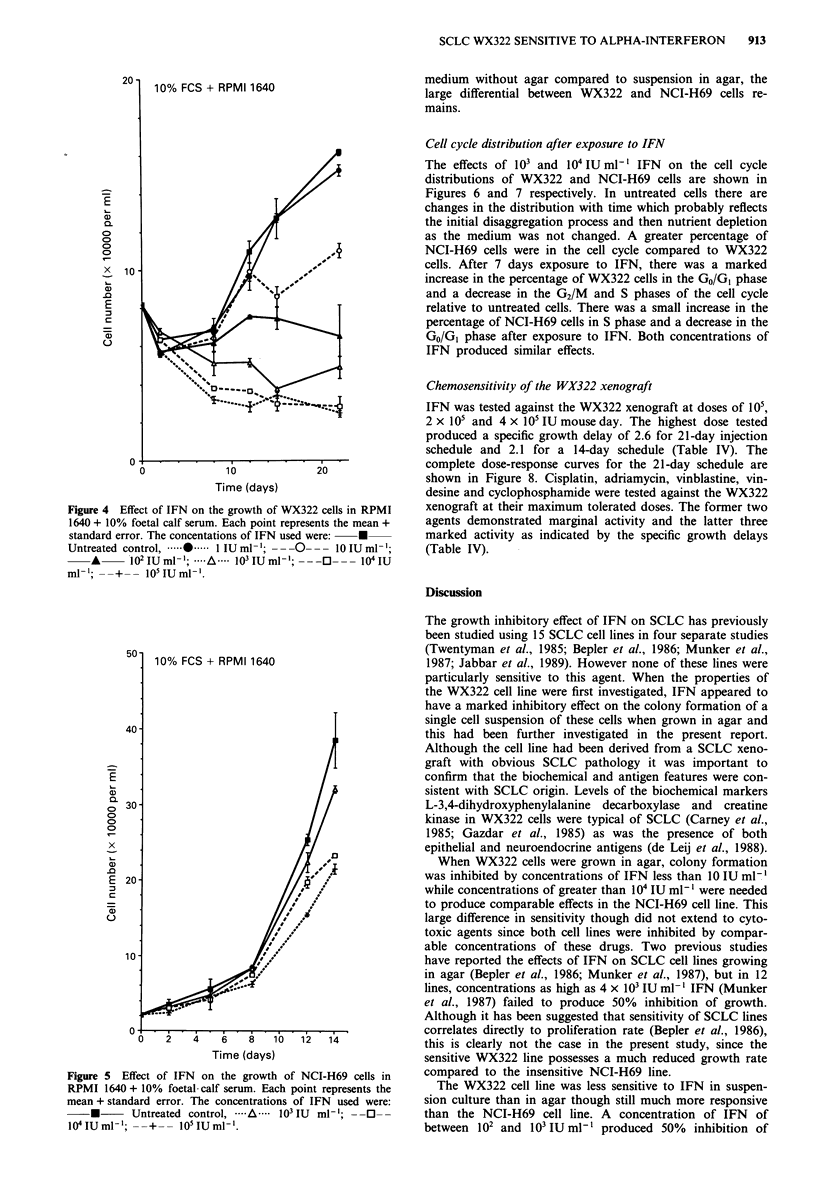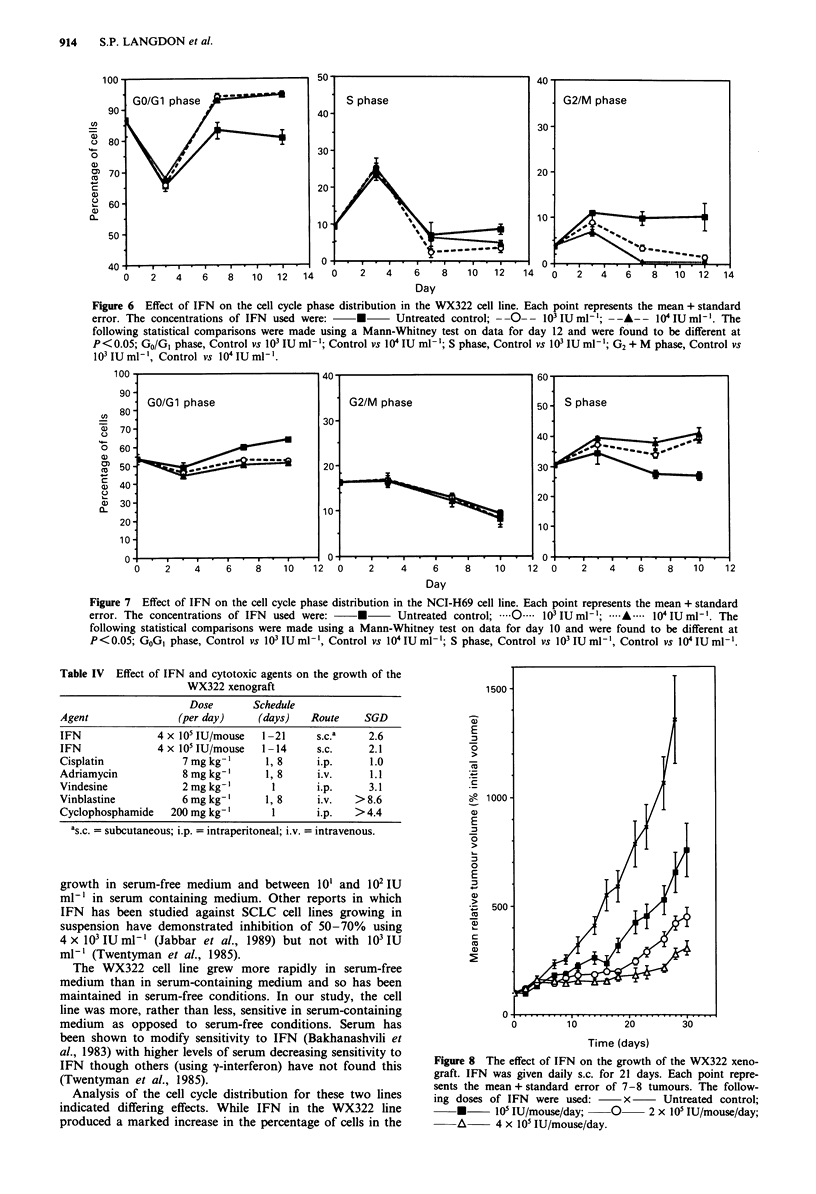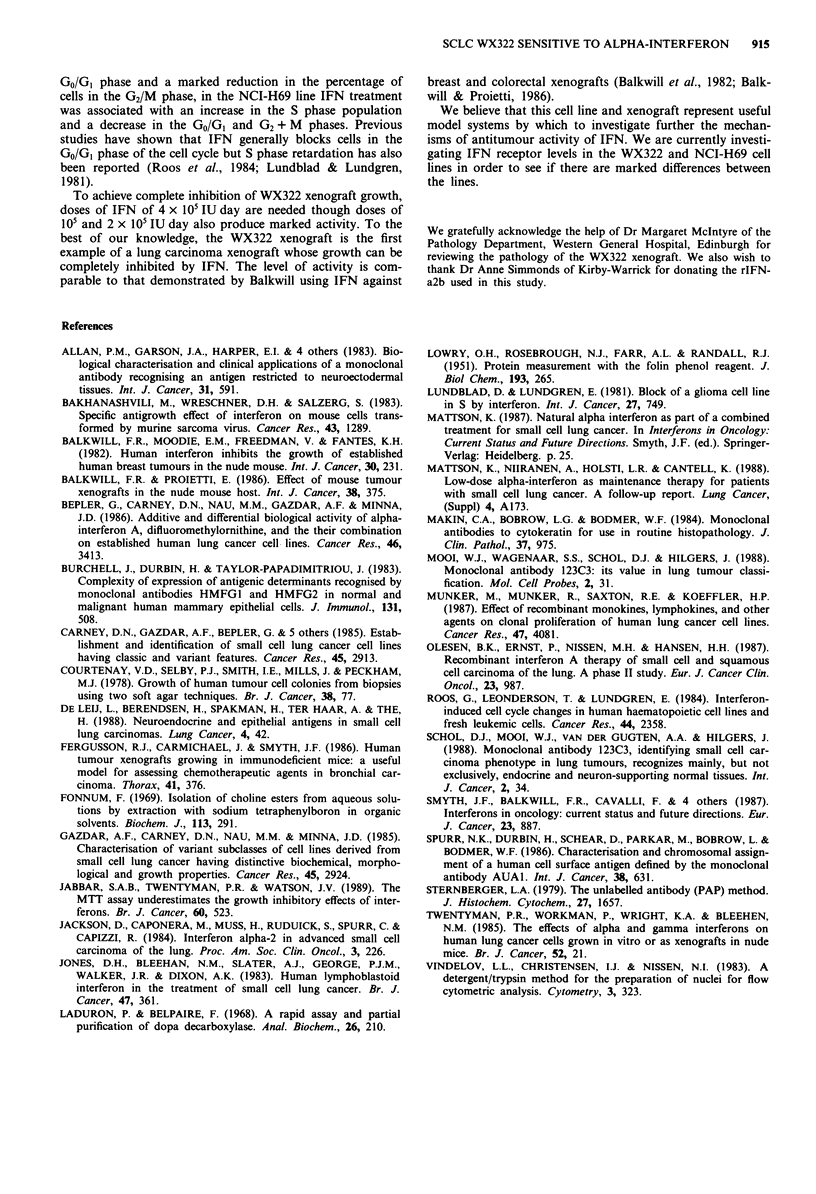# Characterisation and properties of a small cell lung cancer cell line and xenograft WX322 with marked sensitivity to alpha-interferon.

**DOI:** 10.1038/bjc.1991.200

**Published:** 1991-06

**Authors:** S. P. Langdon, G. J. Rabiasz, L. Anderson, A. A. Ritchie, R. J. Fergusson, F. G. Hay, E. P. Miller, P. Mullen, J. Plumb, W. R. Miller

**Affiliations:** ICRF Medical Oncology Unit, Western General Hospital, Edinburgh, UK.

## Abstract

**Images:**


Br. J. Cancer (1991), 63, 909-915 © Macmillan Press Ltd., 1991

### Characterisation and properties of a small cell lung cancer cell line and xenograft WX322 with marked sensitivity to alpha-interferon

S.P. Langdon[1], G.J. Rabiasz[1], L. Anderson[1], A.A. Ritchie[1], R.J. Fergusson[1], F.G. Hay[1], E.P. Miller[1], P. Mullen[1], J. Plumb[2], W.R. Miller[1] & J.F. Smyth[1]

[1]*ICRF Medical Oncology Unit, Western General Hospital, Edinburgh EH4 2XU;* [2]*CRC Department of Medical Oncology, Alexander Stone Building, Garscube Estate, Glasgow G61 1BD, UK.*

**Summary** Controversy exists as to whether interferons usefully influence the growth of epithelial carcinomas. A small cell lung carcinoma (SCLC) cell line, WX322, has been dervied which is $>1000$-fold more sensitive to alpha-interferon (IFN) when grown in agar than other reported SCLC cell lines. The WX322 line has been characterised to prove its epithelial origin and its chemosensitivity compared with that of the NCI-H69 small cell line. The WX322 cell line expresses neuroendocrine and epithelial markers and possesses a morphology consistent with SCLC origin. A concentration of $5\ \mathrm{IU\ ml^{-1}}$ of IFN produced 50% inhibition of colony formation in agar in the WX322 line, whereas a concentration of greater than $10^5\ \mathrm{IU\ ml^{-1}}$ was required to produce a comparable effect with the NCI-H69 cell line. In contrast, WX322, possessed similar sensitivity to NCI-H69 cells when exposed to a range of cytotoxic agents. Analysis of the cell cycle indicated that IFN increased the percentage of cells in the $G_0/G_1$ phase for the WX322 cell line but increased the percentage in S phase for the NCI-H69 line. Growth of the xenograft, from which the cell line was derived, was also inhibited by IFN at doses greater than $10^5$ IU/mouse/day. The WX322 cell line whether grown in agar or as a xenograft shows an unusually high sensitivity to IFN and provides an interesting model for studying mechanisms of IFN cytotoxicity to epithelial cells.

Clinical studies have indicated that various leukaemias and lymphomas are responsive to alpha-interferon (IFN) (Smyth *et al.*, 1987). In contrast, epithelial tumours are generally unresponsive to this agent unless it is administered locally. In the case of small cell lung carcinoma (SCLC), the Phase II studies using IFN have been largely negative (Olesen *et al.*, 1987; Jones *et al.*, 1983; Jackson *et al.*, 1984; Mattson, 1987). However, an ongoing Phase III study exploring the use of IFN as maintenance therapy for SCLC (after initial response was obtained with chemo- and radiotherapy) is suggesting a trend towards long term survival compared to no treatment or treatment with maintenance chemotherapy (Mattson *et al.*, 1988; Mattson, 1987). These results suggest that IFN may have a role as adjuvant therapy in a subset of epithelial tumours. It is therefore of interest that while several groups have examined the growth modifying effects of IFN on cell lines derived from SCLC (Twentyman *et al.*, 1985; Bepler *et al.*, 1986; Munker *et al.*, 1987; Jabbar *et al.*, 1989), none have reported on marked sensitivity to IFN.

However, we here describe a new cell line, WX322, the growth of which in agar is approximately 1000-fold more sensitive to IFN than that for previously described SCLC cell lines. The cell line has been characterised to confirm its human origin and SCLC derivation and its sensitivity to other cytotoxic agents determined. The cell line has also been established in immunosuppressed mice and its sensitivity to IFN has been investigated.

### Materials and methods

### *Origin of the tumour*

The original tumour material was obtained in 1985 from a subcutaneous deposit in a 64-year old man suffering from metastatic small cell lung carcinoma (SCLC). The patient had not been treated previously with any form of therapy. The tumour was initially implanted as a xenograft in CBA mice immunosuppressed by thymectomy and whole body irradiation (Fergusson *et al.*, 1986) and maintained in nude mice from 1988 onwards. Histological analysis confirmed the pathology of SCLC in the patient tumour and the pathology of the xenograft was checked at each passage and has not changed over 5 years.

### *Initiation of the cell line*

The cell line was derived from the xenograft at the 8th passage. The tumour was disaggregated into small fragments with a scalpel and suspended in RPMI 1640 supplemented with hydrocortisone (10 nM), insulin ($5\ \mu g\ ml^{-1}$), transferrin ($10\ \mu g\ ml^{-1}$), sodium selenite (30 nM), glutamine (2 mM), penicillin ($100\ \mathrm{IU\ ml^{-1}}$) and 3-[N-morpholino] propane sulfonic acid (12.5 mM) (referred to as RPMI + HITS). These cells were then cultured at 37°C, 90% humidity and 5% $CO_2$. Although cultures were initially contaminated with stromal fibroblasts and macrophages, these adhered to plastic and so could be readily separated from the suspension cultures within the first two passages. Once established, cultures were routinely passaged by a 1:5 to 1:10 split every 2 weeks. Routine assays for mycoplasma were carried out and found to be negative. The present studies were conducted on cells between their 6th and 20th passage. The NCI-H69 cell line was kindly supplied by Prof. A. Harris, Newcastle and used at passages of between 65 and 75.

### *Estimation of L-3,4-dihydroxyphenylalanine decarboxylase activity*

L-3,4-dihydroxyphenylalanine decarboxylase activity was estimated by a modification of the method of Laduron and Belpaire (1968). Cells were centrifuged at 200 *g* for 5 min and the pellet washed twice with phosphate buffered saline. The pellet was resuspended in borate buffer (0.025 M, pH 7.6) and the cells lysed by freezing at −70°C and thawing at 37°C three times. The lysate was mixed with pyridoxal-5-phosphate (final concentration of 400 $\mu$M). Enzyme activity was measured as the rate of conversion of $^3$H L-3,4-dihydroxyphenylalanine to $^3$H L-dopamine and the $^3$H L-dopamine is separated from $^3$H L-3,4-dihydroxyphenylalanine by liquid cation exchange (Fonnum, 1969). Activity was expressed as International Units per mg protein where 1 unit was equal to 1 $\mu$mol of dopamine formed per minute under the specified reaction conditions. Protein concentration was determined by the method of Lowry *et al.* (1951).

Correspondence: S.P. Langdon.
Received 19 October 1990; and in revised form 16 January 1991.

*Estimation of creatine kinase activity*

Creatine kinase activity was determined spectrophotometrically using a kit (CK reagent, Sigma Diagnostics, Poole, Dorset). Cell lysates were prepared as for the L-3,4-dihydroxyphenylalanine decarboxylase assay. Activity is expressed as units per mg protein where one unit is defined as the amount of enzyme which produces one micromole of NADH per minute from a glucose-6-phosphate dehydrogenase linked reaction under the specified conditions.

The isoenzyme profile was determined electrophoretically using a kit (Cardiotrak-CK, Corning Diagnostics Ltd, Halstead, Essex). The relative amounts of the three isoenzymes was determined with a scanning fluorimeter (Helena Densitometer, Beaumont, Texas, USA).

*Electron microscopy*

For transmission electron microscopy, the tumour cells were pelleted and incubated in 2% glutaraldehyde in phosphate buffered saline. Post fixation was performed in 2% osmium tetroxide in phosphate buffered saline. The material was embedded in Epon.

*Measurement of chemosensitivity in agar*

The soft agar assay of Courtenay *et al.* (1978) was used without the addition of irradiated feeder cells. Cells were placed into semi-liquid culture in agar (0.3% v/v) in round bottom tubes (Falcon 2051) with or without drug. A cell density of $2 \times 10^4$/tube was used for WX322 and $1 \times 10^3$/tube for NCI-H69 as these densities produce approximately 100 colonies/tube. Fresh medium (RPMI + 10% FCS, 1 ml/tube) was added each week. After 28 days, the agar plug in each tube was transferred to a petri dish and colonies (> 50 cells) counted using a microscope.

The drugs were obtained from the following sources: adriamycin (Farmitalia Carlo Erba, St Albans, UK), cisplatin and 5-fluorouracil (DBL, Warwick, UK), vindesine (Eli Lilly, Basingstoke, UK), TCNU (Leo Laboratories, Helsinburg, Sweden). rIFN-a2b was a kind gift from Dr A. Simmonds at Kirby Warrick, Bury St Edmonds, UK.

*Growth experiments in suspension culture*

Cells were suspended in 24 well dishes (Gibco, Paisley, Scotland) at a density of $6-8 \times 10^4$ $ml^{-1}$ for WX322 and at a density of $2 \times 10^4$ $ml^{-1}$ for NCI-H69 in either RPMI 1640 + HITS (described above) or RPMI 1640 + 10% FCS. IFN was added at the same time at concentrations ranging from 1 to 100,000 IU $ml^{-1}$. On the days shown in Figures 2–4, the cell suspension was removed from groups of wells. Cell clusters were disaggregated by passage several times through a 19 gauge needle (Becton, Dickinson and Co, Dublin, Eire) prior to counting in a model ZF Coulter Counter.

*Immunoperoxidase staining*

The immunohistochemical studies of the cultured cells were performed according to the peroxidase anti-peroxidase method as follows (Sternberger, 1979). Cells were placed onto multispot slides (Hendley [Essex] Ltd, Essex, UK) at approximately $2 \times 10^4$ cells/spot and fixed in methanol: acetone (1:1) for 10 min. The fixed preparations were then incubated at room temperature with hydrogen peroxide (0.5% in methanol) for 15 min to block endogenous peroxidase, washed in Tris buffered saline (TBS, Tris 0.5 M, pH 7.6 diluted in saline 1:10) and successively incubated with sheep serum: TBS (1:4) for 10 min and an appropriate dilution (described below) of the mouse MoAb for 30 min. Thereafter sheep anti-mouse IgG (SAPU, Carluke, UK) was applied at a dilution of 1:5 in TBS for 30 min at room temperature. After being washed with TBS, these samples were incubated with mouse monoclonal PAP complex (Dako Ltd, High Wycombe, UK) at optimal dilution (1:200) in TBS. Finally the peroxidase was localised by treatment of the samples with a fresh mixture of 3,3'-diaminobenzidine (0.1%) and hydrogen peroxide (0.1%) in Tris-imidazole buffer (pH 7.6) for 10 min, and after washing with water, these samples were counterstained with hematoxylin.

MoAbs were obtained from the following sources and used at these dilutions. 123C3 and 123A8 were gifts from Dr J. Hilgers, Netherlands Cancer Institute, Amsterdam and were used at a dilution of 1:100 of the ascites (Schol *et al.*, 1988; Mooi *et al.*, 1988). CAM 5.2 (Makin *et al.*, 1984), AUA1 (Spurr *et al.*, 1986), UJ13A (Allan *et al.*, 1983), HMFG1 and HMFG2 (Burchell *et al.*, 1983) were gifts from ICRF, London and were used as supernatants. Leukocyte common antigen (DAKO-LC), used as a negative control throughout, was obtained from DAKO and used as a 1:20 dilution of supernatant.

*Cell cycle phase distribution after exposure to IFN*

Cells were set up in 6-well plates with or without $10^3$ or $10^4$ IU $ml^{-1}$ IFN. Samples of approximately $10^6$ cells were prepared from quadruplicate wells at each time point for DNA analysis by a trypsin/detergent method using propidium iodide as a stain (Vindelov *et al.*, 1983). Analysis was carried out using a FACScan flow cytometer equipped for doublet discrimination (Becton Dickinson) using Cellfit software. All data was gated on forward and side scatter signals to exclude fragmented and clumped material and on a fluorescence width *vs* fluorescence area signal to exclude doublets. Cell cycle distribution analysis was performed using the RFIT model which calculates S phase as a rectangle bounded by the mean $G_0/G_1$ and $G_2/M$ channels with a height of the mean S phase channel. The mean coefficient of variation of the $G_0/G_1$ histogram was 4.8.

*In vivo testing*

Female nu/nu (nude) mice (originally bred at ICRF Laboratories, London) were obtained from OLAC Ltd, and maintained in negative pressure isolators (La Calhene, Cambridge). The WX322 xenograft was maintained as a subcutaneous tumour in the flank of these mice and used at passages 25–28 for the experiments described. In these experiments, fragments of the WX322 xenograft (obtained from passage animals) were implanted subcutenously into the flanks of animals. After approximately 1 month, when tumours reached a volume of 100–200 $mm^3$, animals were randomly allocated to treatment or saline control groups (each of six animals) and treatment commenced (defined as Day 0). The doses and schedules used for each agent are described in Table IV. Tumours were measured three times a week using vernier calipers. Tumour volumes were estimated by using the formula: volume (V) = $\pi/6 \times l \times w^2$ where l is the longest diameter and w is the diameter perpandicular to this.

The relative tumour volume, $V_t/V_o$ (where $V_o$ is the tumour volume at the start of treatment and $V_t$ is the tumour volume at any given time), was calculated for each individual tumour at every time point. The specific growth delay (SGD) of a treated group as compared to controls was calculated by the following formula:

$$SGD = \frac{T_D \text{ (treated group)} - T_D \text{ (control group)}}{T_D \text{ (control group)}}$$

where $T_D$ = Tumour doubling time i.e. time for group to reach a median relative tumour volume of 200%.

### Results

*Morphology of the cell line*

The WX322 cell line grows as loose clusters of cells (Figure 1a) with an appearance intermediate between Carney's Type 2 and Type 3 descriptions of SCLC cell lines (Carney *et al.*,

**Figure 1** **a**, Photomicrograph of WX322 cells in suspension (× 625), **b**, Electron micrograph of WX322 cells showing desmosomes (indicated by arrows) between two cells (× 50,000), **c**, Section of the WX322 xenograft demonstrating typical SCLC pathology (× 1563).

1985). Electron microscopy revealed the presence of desmosomes consistent with an epithelial origin (Figure 1b), though dense core granules were not observed. The xenograft from which the cell line was initiated possessed a pathology typical of SCLC (Figure 1c).

*Biochemical and immunohistochemical properties of the cell line*

The SCLC markers L-3,4-dihydroxyphenylalanine decarboxylase and creatine kinase BB were both found in the WX322 cell line (Table I). Comparison with the classic NCI-H69 cell line indicated that levels of L-3,4-dihydroxyphenylalanine decarboxylase were similar in the two lines while levels of creatine kinase in the WX322 cells were approximately 1/3 level of H69 cells (Table I). The isoenzyme profile for the various forms of creatine kinase was similar for the two cell lines (Table I). WX322 cells were stained with a number of

**Table I** Enzyme content of the cell lines

| | *WX322* | *NCI-H69* |
|---|---|---|
| L-3,4-dihydroxyphenylalanine decarboxylase (IU $mg^{-1}$ protein) | 2346±42[a] | 3734±111 |
| Creatine kinase (U $mg^{-1}$ protein) | 0.67 ± 0.02 | 2.22±0.34 |
| Creatine kinase (%) | | |
| MM | 7.3 | 4.8 |
| MB | 0 | 0 |
| BB | 92.7 | 95.2 |

[a]Mean±standard deviation of three measurements.

Table II Antigen expression of WX322 cells

| MoAb | Antigen type | Antigen detected | (% cells + ve)[a] |
|---|---|---|---|
| HMFG1 | Epithelial | Human milk fat globule mem- | >90 |
| HMFG2 | Epithelial | brane | >90 |
| AUA1 | Epithelial | 35 kd protein | >90 |
| CAM 5.2 | Epithelial | Cytokeratins 8, 18 and 19 | 18±5 |
| 123A8 | Neuroendocrine | Neural cell adhesion molecule | >90 |
| 123C3 | Neuroendocrine | Neural cell adhesion molecule | >90 |
| UJ13A | Neuroendocrine | Neural cell adhesion molecule | 25±10 |

[a]Mean±standard deviation of four separate experiments.

monoclonal antibodies which detect either epithelial or neuroendocrine antigens, both of which are commonly found in SCLC cells. Both types of marker were expressed in the majority of these cells (Table II). Thus more than 90% WX322 cells stained positively with the epithelial antibodies HMFG1, HMFG2 and AUAI and the neuroendocrine markers 123C3 and 123A8 while CAM 5.2 reacted with about 18% of cells and UJ13A with 25%. This expression of surface antigens in WX322 cells is consistent with that of an SCLC line.

*Chemosensitivity of the WX322 and NCI-H69 cell lines in agar suspension*

The sensitivity of the WX322 and H69 cell lines to IFN and several antitumour agents used clinically for the treatment of SCLC were examined using an agar clonogenic assay. Concentrations producing 50% inhibition of colony formation are compared in Table III. The WX322 cell line was over 20,000-fold more sensitive to IFN than the NCI–H69 cell line. Thus the $IC_{50}$ for the WX322 line was 5 IU $ml^{-1}$ IFN compared to greater than $10^5$ IU $ml^{-1}$ for the NCI-H69 line. The dose-response curves for exposure of these two lines to IFN are shown in Figure 2. In contrast, the two cell lines show similar sensitivity to the cytotoxic agents cisplatin, adriamycin, 5-fluorouracil, TCNU and vindesine.

*Sensitivity of the WX322 and NCI-H69 cell lines to IFN in suspension without agar*

Growth curves for the WX322 cell line with IFN in either serum-free conditions (RPMI 1640 + HITS) or serum-containing conditions (RPMI 1640 + 10% FCS) are shown in Figures 3 and 4. NCI-H69 cells grew very poorly in serum-free conditions (data not shown) and data for NCI-H69 cells with IFN in the presence of serum is shown in Figure 5. Concentrations greater than 10 IU $ml^{-1}$ inhibited cell growth of WX322 cells in either serum-free or serum-containing media while a concentration greater than 1000 IU $ml^{-1}$ was needed to inhibit growth of the NCI-H69 cell line in serum-containing medium. Increasing the IFN concentration above these values produced increasing inhibition indicating a concentration-response relationship. Concentrations of $10^4$ IU $ml^{-1}$ in serum-free conditions and $10^2$ IU $ml^{-1}$ in serum-containing conditions were cytostatic for WX322 cells while a concentration greater than $10^5$ IU $ml^{-1}$ would be required to produce cytostasis for NCI-H69 cells. Therefore while the WX322 line appears less sensitive to IFN when suspended in

**Figure 2** Effect of IFN on the colony formation in agar of the WX322 and NCI-H69 SCLC lines. Mean colony number + standard error indicated. ——■—— WX322; ——□—— H69. Each point represents the mean of at least three separate experiments.

**Figure 3** Effect of IFN on the growth of WX322 cells in serum-free conditions (HITS + RPMI 1640). Each point represents the mean + standard error. The concentrations of IFN used were: ——■—— Untreated control, ····●···· 1 IU $ml^{-1}$; – –O– – 10 IU $ml^{-1}$; ——▲—— $10^2$ IU $ml^{-1}$; ····Δ···· $10^3$ IU $ml^{-1}$; – –□– – $10^4$ IU $ml^{-1}$; – –+– – $10^5$ IU $ml^{-1}$.

Table III Sensitivity of the WX322 and NCI-H69 cell lines in agar to cytotoxic agents and IFN

| | $IC_{50}$ Concentration[a] | | |
|---|---|---|---|
| Agent | WX322 | NCI-H69 | $IC_{50}$ ratio |
| IFN | 5 IU $ml^{-1}$ | > 100,000 IU $ml^{-1}$ | > 20,000 |
| Cisplatin | 0.06 μM | 0.22 μM | 3.7 |
| Adriamycin | 7.5 nM | 10.2 nM | 1.4 |
| 5-Fluorouracil | 1.5 μM | 3.2 μM | 2.1 |
| TCNU | 5 μM | 3 μM | 0.6 |
| Vindesine | 0.5 nM | 0.5 nM | 1.0 |

[a]$IC_{50}$ = 50% inhibition of colony formation. The value shown is the mean of three separate experiments.

**Figure 4** Effect of IFN on the growth of WX322 cells in RPMI 1640 + 10% foetal calf serum. Each point represents the mean + standard error. The concentations of IFN used were: ——■—— Untreated control, ·····●····· 1 IU ml$^{-1}$; - - -O- - - 10 IU ml$^{-1}$; ——▲—— $10^2$ IU ml$^{-1}$; ····△···· $10^3$ IU ml$^{-1}$; - - -□- - - $10^4$ IU ml$^{-1}$; - -+- - $10^5$ IU ml$^{-1}$.

**Figure 5** Effect of IFN on the growth of NCI-H69 cells in RPMI 1640 + 10% foetal calf serum. Each point represents the mean + standard error. The concentrations of IFN used were: ——■—— Untreated control, ····△···· $10^3$ IU ml$^{-1}$; - -□- - $10^4$ IU ml$^{-1}$; - -+- - $10^5$ IU ml$^{-1}$.

medium without agar compared to suspension in agar, the large differential between WX322 and NCI-H69 cells remains.

*Cell cycle distribution after exposure to IFN*

The effects of $10^3$ and $10^4$ IU ml$^{-1}$ IFN on the cell cycle distributions of WX322 and NCI-H69 cells are shown in Figures 6 and 7 respectively. In untreated cells there are changes in the distribution with time which probably reflects the initial disaggregation process and then nutrient depletion as the medium was not changed. A greater percentage of NCI-H69 cells were in the cell cycle compared to WX322 cells. After 7 days exposure to IFN, there was a marked increase in the percentage of WX322 cells in the $G_0/G_1$ phase and a decrease in the $G_2/M$ and S phases of the cell cycle relative to untreated cells. There was a small increase in the percentage of NCI-H69 cells in S phase and a decrease in the $G_0/G_1$ phase after exposure to IFN. Both concentrations of IFN produced similar effects.

*Chemosensitivity of the WX322 xenograft*

IFN was tested against the WX322 xenograft at doses of $10^5$, $2 \times 10^5$ and $4 \times 10^5$ IU mouse day. The highest dose tested produced a specific growth delay of 2.6 for 21-day injection schedule and 2.1 for a 14-day schedule (Table IV). The complete dose-response curves for the 21-day schedule are shown in Figure 8. Cisplatin, adriamycin, vinblastine, vindesine and cyclophosphamide were tested against the WX322 xenograft at their maximum tolerated doses. The former two agents demonstrated marginal activity and the latter three marked activity as indicated by the specific growth delays (Table IV).

### Discussion

The growth inhibitory effect of IFN on SCLC has previously been studied using 15 SCLC cell lines in four separate studies (Twentyman *et al.*, 1985; Bepler *et al.*, 1986; Munker *et al.*, 1987; Jabbar *et al.*, 1989). However none of these lines were particularly sensitive to this agent. When the properties of the WX322 cell line were first investigated, IFN appeared to have a marked inhibitory effect on the colony formation of a single cell suspension of these cells when grown in agar and this had been further investigated in the present report. Although the cell line had been derived from a SCLC xenograft with obvious SCLC pathology it was important to confirm that the biochemical and antigen features were consistent with SCLC origin. Levels of the biochemical markers L-3,4-dihydroxyphenylalanine decarboxylase and creatine kinase in WX322 cells were typical of SCLC (Carney *et al.*, 1985; Gazdar *et al.*, 1985) as was the presence of both epithelial and neuroendocrine antigens (de Leij *et al.*, 1988).

When WX322 cells were grown in agar, colony formation was inhibited by concentrations of IFN less than 10 IU ml$^{-1}$ while concentrations of greater than $10^4$ IU ml$^{-1}$ were needed to produce comparable effects in the NCI-H69 cell line. This large difference in sensitivity though did not extend to cytotoxic agents since both cell lines were inhibited by comparable concentrations of these drugs. Two previous studies have reported the effects of IFN on SCLC cell lines growing in agar (Bepler *et al.*, 1986; Munker *et al.*, 1987), but in 12 lines, concentrations as high as $4 \times 10^3$ IU ml$^{-1}$ IFN (Munker *et al.*, 1987) failed to produce 50% inhibition of growth. Although it has been suggested that sensitivity of SCLC lines correlates directly to proliferation rate (Bepler *et al.*, 1986), this is clearly not the case in the present study, since the sensitive WX322 line possesses a much reduced growth rate compared to the insensitive NCI-H69 line.

The WX322 cell line was less sensitive to IFN in suspension culture than in agar though still much more responsive than the NCI-H69 cell line. A concentration of IFN of between $10^2$ and $10^3$ IU ml$^{-1}$ produced 50% inhibition of

**Figure 6** Effect of IFN on the cell cycle phase distribution in the WX322 cell line. Each point represents the mean + standard error. The concentrations of IFN used were: ——■—— Untreated control; --O-- $10^3$ IU ml$^{-1}$; --▲-- $10^4$ IU ml$^{-1}$. The following statistical comparisons were made using a Mann-Whitney test on data for day 12 and were found to be different at $P<0.05$; $G_0/G_1$ phase, Control *vs* $10^3$ IU ml$^{-1}$; Control *vs* $10^4$ IU ml$^{-1}$; S phase, Control *vs* $10^3$ IU ml$^{-1}$; $G_2$ + M phase, Control *vs* $10^3$ IU ml$^{-1}$, Control *vs* $10^4$ IU ml$^{-1}$.

**Figure 7** Effect of IFN on the cell cycle phase distribution in the NCI-H69 cell line. Each point represents the mean + standard error. The concentrations of IFN used were: ——■—— Untreated control; ····O···· $10^3$ IU ml$^{-1}$; ····▲···· $10^4$ IU ml$^{-1}$. The following statistical comparisons were made using a Mann-Whitney test on data for day 10 and were found to be different at $P<0.05$; $G_0G_1$ phase, Control *vs* $10^3$ IU ml$^{-1}$, Control *vs* $10^4$ IU ml$^{-1}$; S phase, Control *vs* $10^3$ IU ml$^{-1}$, Control *vs* $10^4$ IU ml$^{-1}$.

**Table IV** Effect of IFN and cytotoxic agents on the growth of the WX322 xenograft

| *Agent* | *Dose (per day)* | *Schedule (days)* | *Route* | *SGD* |
|---|---|---|---|---|
| IFN | 4 × $10^5$ IU/mouse | 1–21 | s.c.[a] | 2.6 |
| IFN | 4 × $10^5$ IU/mouse | 1–14 | s.c. | 2.1 |
| Cisplatin | 7 mg kg$^{-1}$ | 1, 8 | i.p. | 1.0 |
| Adriamycin | 8 mg kg$^{-1}$ | 1, 8 | i.v. | 1.1 |
| Vindesine | 2 mg kg$^{-1}$ | 1 | i.p. | 3.1 |
| Vinblastine | 6 mg kg$^{-1}$ | 1, 8 | i.v. | >8.6 |
| Cyclophosphamide | 200 mg kg$^{-1}$ | 1 | i.p. | >4.4 |

[a]s.c. = subcutaneous; i.p. = intraperitoneal; i.v. = intravenous.

growth in serum-free medium and between $10^1$ and $10^2$ IU ml$^{-1}$ in serum containing medium. Other reports in which IFN has been studied against SCLC cell lines growing in suspension have demonstrated inhibition of 50–70% using 4 × $10^3$ IU ml$^{-1}$ (Jabbar *et al.*, 1989) but not with $10^3$ IU ml$^{-1}$ (Twentyman *et al.*, 1985).

The WX322 cell line grew more rapidly in serum-free medium than in serum-containing medium and so has been maintained in serum-free conditions. In our study, the cell line was more, rather than less, sensitive in serum-containing medium as opposed to serum-free conditions. Serum has been shown to modify sensitivity to IFN (Bakhanashvili *et al.*, 1983) with higher levels of serum decreasing sensitivity to IFN though others (using γ-interferon) have not found this (Twentyman *et al.*, 1985).

Analysis of the cell cycle distribution for these two lines indicated differing effects. While IFN in the WX322 line produced a marked increase in the percentage of cells in the

**Figure 8** The effect of IFN on the growth of the WX322 xenograft. IFN was given daily s.c. for 21 days. Each point represents the mean + standard error of 7–8 tumours. The following doses of IFN were used: ——×—— Untreated control; ——■—— $10^5$ IU/mouse/day; ——O—— 2 × $10^5$ IU/mouse/day; ——Δ—— 4 × $10^5$ IU/mouse/day.

$G_0/G_1$ phase and a marked reduction in the percentage of cells in the $G_2/M$ phase, in the NCI-H69 line IFN treatment was associated with an increase in the S phase population and a decrease in the $G_0/G_1$ and $G_2 + M$ phases. Previous studies have shown that IFN generally blocks cells in the $G_0/G_1$ phase of the cell cycle but S phase retardation has also been reported (Roos *et al.*, 1984; Lundblad & Lundgren, 1981).

To achieve complete inhibition of WX322 xenograft growth, doses of IFN of $4 \times 10^5$ IU day are needed though doses of $10^5$ and $2 \times 10^5$ IU day also produce marked activity. To the best of our knowledge, the WX322 xenograft is the first example of a lung carcinoma xenograft whose growth can be completely inhibited by IFN. The level of activity is comparable to that demonstrated by Balkwill using IFN against breast and colorectal xenografts (Balkwill *et al.*, 1982; Balkwill & Proietti, 1986).

We believe that this cell line and xenograft represent useful model systems by which to investigate further the mechanisms of antitumour activity of IFN. We are currently investigating IFN receptor levels in the WX322 and NCI-H69 cell lines in order to see if there are marked differences between the lines.

We gratefully acknowledge the help of Dr Margaret McIntyre of the Pathology Department, Western General Hospital, Edinburgh for reviewing the pathology of the WX322 xenograft. We also wish to thank Dr Anne Simmonds of Kirby-Warrick for donating the rIFN-a2b used in this study.

### References

ALLAN, P.M., GARSON, J.A., HARPER, E.I. & 4 others (1983). Biological characterisation and clinical applications of a monoclonal antibody recognising an antigen restricted to neuroectodermal tissues. *Int. J. Cancer*, **31**, 591.

BAKHANASHVILI, M., WRESCHNER, D.H. & SALZERG, S. (1983). Specific antigrowth effect of interferon on mouse cells transformed by murine sarcoma virus. *Cancer Res.*, **43**, 1289.

BALKWILL, F.R., MOODIE, E.M., FREEDMAN, V. & FANTES, K.H. (1982). Human interferon inhibits the growth of established human breast tumours in the nude mouse. *Int. J. Cancer*, **30**, 231.

BALKWILL, F.R. & PROIETTI, E. (1986). Effect of mouse tumour xenografts in the nude mouse host. *Int. J. Cancer*, **38**, 375.

BEPLER, G., CARNEY, D.N., NAU, M.M., GAZDAR, A.F. & MINNA, J.D. (1986). Additive and differential biological activity of alpha-interferon A, difluoromethylornithine, and the their combination on established human lung cancer cell lines. *Cancer Res.*, **46**, 3413.

BURCHELL, J., DURBIN, H. & TAYLOR-PAPADIMITRIOU, J. (1983). Complexity of expression of antigenic determinants recognised by monoclonal antibodies HMFG1 and HMFG2 in normal and malignant human mammary epithelial cells. *J. Immunol.*, **131**, 508.

CARNEY, D.N., GAZDAR, A.F., BEPLER, G. & 5 others (1985). Establishment and identification of small cell lung cancer cell lines having classic and variant features. *Cancer Res.*, **45**, 2913.

COURTENAY, V.D., SELBY, P.J., SMITH, I.E., MILLS, J. & PECKHAM, M.J. (1978). Growth of human tumour cell colonies from biopsies using two soft agar techniques. *Br. J. Cancer*, **38**, 77.

DE LEIJ, L., BERENDSEN, H., SPAKMAN, H., TER HAAR, A. & THE, H. (1988). Neuroendocrine and epithelial antigens in small cell lung carcinomas. *Lung Cancer*, **4**, 42.

FERGUSSON, R.J., CARMICHAEL, J. & SMYTH, J.F. (1986). Human tumour xenografts growing in immunodeficient mice: a useful model for assessing chemotherapeutic agents in bronchial carcinoma. *Thorax*, **41**, 376.

FONNUM, F. (1969). Isolation of choline esters from aqueous solutions by extraction with sodium tetraphenylboron in organic solvents. *Biochem. J.*, **113**, 291.

GAZDAR, A.F., CARNEY, D.N., NAU, M.M. & MINNA, J.D. (1985). Characterisation of variant subclasses of cell lines derived from small cell lung cancer having distinctive biochemical, morphological and growth properties. *Cancer Res.*, **45**, 2924.

JABBAR, S.A.B., TWENTYMAN, P.R. & WATSON, J.V. (1989). The MTT assay underestimates the growth inhibitory effects of interferons. *Br. J. Cancer*, **60**, 523.

JACKSON, D., CAPONERA, M., MUSS, H., RUDUICK, S., SPURR, C. & CAPIZZI, R. (1984). Interferon alpha-2 in advanced small cell carcinoma of the lung. *Proc. Am. Soc. Clin. Oncol.*, **3**, 226.

JONES, D.H., BLEEHAN, N.M., SLATER, A.J., GEORGE, P.J.M., WALKER, J.R. & DIXON, A.K. (1983). Human lymphoblastoid interferon in the treatment of small cell lung cancer. *Br. J. Cancer*, **47**, 361.

LADURON, P. & BELPAIRE, F. (1968). A rapid assay and partial purification of dopa decarboxylase. *Anal. Biochem.*, **26**, 210.

LOWRY, O.H., ROSEBROUGH, N.J., FARR, A.L. & RANDALL, R.J. (1951). Protein measurement with the folin phenol reagent. *J. Biol Chem.*, **193**, 265.

LUNDBLAD, D. & LUNDGREN, E. (1981). Block of a glioma cell line in S by interferon. *Int. J. Cancer*, **27**, 749.

MATTSON, K. (1987). Natural alpha interferon as part of a combined treatment for small cell lung cancer. In *Interferons in Oncology: Current Status and Future Directions*. Smyth, J.F. (ed.). Springer-Verlag: Heidelberg. p. 25.

MATTSON, K., NIIRANEN, A., HOLSTI, L.R. & CANTELL, K. (1988). Low-dose alpha-interferon as maintenance therapy for patients with small cell lung cancer. A follow-up report. *Lung Cancer*, (Suppl) **4**, A173.

MAKIN, C.A., BOBROW, L.G. & BODMER, W.F. (1984). Monoclonal antibodies to cytokeratin for use in routine histopathology. *J. Clin. Pathol.*, **37**, 975.

MOOI, W.J., WAGENAAR, S.S., SCHOL, D.J. & HILGERS, J. (1988). Monoclonal antibody 123C3: its value in lung tumour classification. *Mol. Cell Probes*, **2**, 31.

MUNKER, M., MUNKER, R., SAXTON, R.E. & KOEFFLER, H.P. (1987). Effect of recombinant monokines, lymphokines, and other agents on clonal proliferation of human lung cancer cell lines. *Cancer Res.*, **47**, 4081.

OLESEN, B.K., ERNST, P., NISSEN, M.H. & HANSEN, H.H. (1987). Recombinant interferon A therapy of small cell and squamous cell carcinoma of the lung. A phase II study. *Eur. J. Cancer Clin. Oncol.*, **23**, 987.

ROOS, G., LEONDERSON, T. & LUNDGREN, E. (1984). Interferon-induced cell cycle changes in human haematopoietic cell lines and fresh leukemic cells. *Cancer Res.*, **44**, 2358.

SCHOL, D.J., MOOI, W.J., VAN DER GUGTEN, A.A. & HILGERS, J. (1988). Monoclonal antibody 123C3, identifying small cell carcinoma phenotype in lung tumours, recognizes mainly, but not exclusively, endocrine and neuron-supporting normal tissues. *Int. J. Cancer*, **2**, 34.

SMYTH, J.F., BALKWILL, F.R., CAVALLI, F. & 4 others (1987). Interferons in oncology: current status and future directions. *Eur. J. Cancer*, **23**, 887.

SPURR, N.K., DURBIN, H., SCHEAR, D., PARKAR, M., BOBROW, L. & BODMER, W.F. (1986). Characterisation and chromosomal assignment of a human cell surface antigen defined by the monoclonal antibody AUA1. *Int. J. Cancer*, **38**, 631.

STERNBERGER, L.A. (1979). The unlabelled antibody (PAP) method. *J. Histochem. Cytochem.*, **27**, 1657.

TWENTYMAN, P.R., WORKMAN, P., WRIGHT, K.A. & BLEEHEN, N.M. (1985). The effects of alpha and gamma interferons on human lung cancer cells grown in vitro or as xenografts in nude mice. *Br. J. Cancer*, **52**, 21.

VINDELOV, L.L., CHRISTENSEN, I.J. & NISSEN, N.I. (1983). A detergent/trypsin method for the preparation of nuclei for flow cytometric analysis. *Cytometry*, **3**, 323.